# The tumor immune microenvironment and T-cell-related immunotherapies in colorectal cancer

**DOI:** 10.1007/s12672-024-01117-7

**Published:** 2024-06-25

**Authors:** Liu Chuang, Ju Qifeng, Yu Shaolei

**Affiliations:** 1 Hanan Branch of the Second Affiliated Hospital of Heilongjiang University of Chinese Medicine, Guogoli Street, Nangang District, Harbin, China; 2 The First Affiliated Hospital Heilongjiang University of Chinese Medicine, Harbin, China

**Keywords:** Colorectal cancer, Tumor immune microenvironment, T cell, Monoclonal antibody, CAR-T

## Abstract

The tumor microenvironment includes a complex network of immune T-cell subsets that play important roles in colorectal cancer (CRC) progression and are key elements of CRC immunotherapy. T cells develop and migrate within tumors, recognizing tumor-specific antigens to regulate immune surveillance. Current immunotherapies are divided into the following main categories based on the regulatory role of T-cell subsets in the tumor immune microenvironment (TIME): cytokines, monoclonal antibodies, peptide vaccines, CAR-T cells and more. This review describes the composition of the tumor immune microenvironment in colorectal cancer and the involvement of T cells in the pathogenesis and progression of CRC as well as current T-cell-related immunotherapies. Further studies on CRC-specific tumor antigens, the gene regulation of T cells, and the regulation of immune activity are needed.

## Introduction

Colorectal cancer (CRC) is one of the most common types of cancer, with more than one million patients diagnosed with CRC each year and more than 900,000 deaths attributed to this disease [1]. The incidence of CRC is greater in males than in females, and the incidence of CRC increases significantly with advanced age [1]. Increasing evidence suggests that the immune system plays a crucial role in the occurrence, growth, and metastasis of CRC. The adaptive immune response at the tumor site plays a pivotal role in balancing tumor invasion and cancer defense [2]. In recent years, cancer immunotherapy has aimed to utilize the intricacy and specificity of the immune system for the treatment of various malignancies, including CRC [3]. Here, we provide an overview of the mechanisms by which T cells regulate the tumor immune microenvironment and summarize the research progress on T-cell-related immunotherapy in CRC.

## The tumor immune microenvironment is composed of a T-cell subset network

The specific composition of the tumor microenvironment varies depending on the type of tumor, but most tumor types share common characteristics, including tumor cells, immune cells, stromal cells, the extracellular matrix, blood vessels, and soluble factors. Within the tumor microenvironment, all immune components are collectively defined as the tumor immune microenvironment (TIME), which has specific internal interactions and plays crucial roles in tumor biology [4]. Dysregulation of immune responses can trigger infiltration and accumulation of immune cells, leading to the release of various cytokines, chemokines, and growth factors, which may further influence the processes of inflammation and carcinogenesis. Therefore, the regulation of each link in TIME may be of great significance for the diagnosis and treatment of tumors.

Previous studies using histological or bulk RNA-Sequence analysis have indicated a correlation between T-cell infiltration and clinical outcomes and survival rates in CRC patients [5–7]. T lymphocytes are composed of different subsets, including regulatory T (Treg) cells, helper T (Th) cells, and cytotoxic T lymphocytes (CTLs). The subsets of helper T cells primarily include Th1, Th2, and Th17 cells, which differentiate from naïve CD4^+^ T cells [8]. By secreting various specific cytokines, Th1 and Th2 cells are involved in cellular immunity and humoral immunity, respectively [9]. Tregs can be classified according to their developmental origin: thymus derived or naturally occurring Treg cells develop in the thymus as separate lineages at the CD4 + single-positive thymocyte stage and are thought to have high affinity for T-cell antigen receptor (TCR) enrichment for their own peptides [10]. Compared to in vitro produced Treg (iTreg) cells, peripherally derived Treg (pTreg) cells were produced peripherally when activated by antigens in the presence of other factors such as IL-2 and TGF-β [10]. Moreover, Th17 cells participate in the pathogenesis of relapse-remitting multiple sclerosis and autoimmune encephalitis through cytokines such as IL-11 and IL-17A, and their pathogenesis is mainly related to autoimmune regulation [11, 12].

Tumor cells undergo a selection process called immunoediting that consists of 3 stages: elimination, equilibrium, and escape. In the first stage, the antitumor immune response eliminates the original tumor cells. The tumor then evolves into a static stage (equilibrium), in which some malignant cells avoid the immune response and do not eliminate the tumor. Finally, resistant clones evade the immune system, acquire tumor-promoting properties and reduce their immunogenicity, thus allowing tumor development and clinical manifestations [13].

The TIME in CRC is heterogeneous. Several studies have shown that Tregs are associated with favorable outcomes in patients with colorectal cancer [14, 15]. In contrast, other studies have reported that Tregs promote tumor progression by suppressing antitumor immunity [16]. Additionally, patients with high expression of the Th17 cluster in colorectal cancer tumor tissues have a worse prognosis than patients with low expression [17]. The results of serum inflammatory cytokine detection in CRC patients have revealed significant upregulation of TGFb1, IL-10, and IL-23, which are associated with Tregs and Th17 cells. Single-cell analysis has showed significant diversity in CRC, with T-bet being expressed not only in Th1 cells but also in Th17 cells and Tregs [18]. All these studies indicate that alterations in T-cell subsets are involved in the progression of CRC and that regulating expression of T-cell-specific genes or inflammatory cytokines may be important therapeutic approaches.

There is molecular heterogeneity in CRC, and differences in prognosis have been observed between patients with the same disease stage; these differences are associated with different genetic mutations [19]. The primary genetic modification of CRC relies on impaired DNA mismatch repair activity, which results in microsatellite instability (MSI) phenotypes in 15% of tumors, unlike the majority of microsatellite stable (MSS) tumors without such damage, which account for 85% of CRC cases [20]. Many research groups have proposed the CRC subtype based on large-scale gene expression studies. The International Consortium has published consensus molecular subtypes (CMS) that classify CRC into CMS1 (MSI immune), CMS2 (classical), CMS3 (metabolic) and CMS4 (mesenchymal) based on a large number of transcriptomic characteristics [21], which further characterized the cellular diversity and heterogeneity within tumor and microenvironment cells. Different phenotypes of CRC exhibit different immune microenvironments; for example, while most CRC patients exhibit poor infiltration of MSS tumor immune cells, the MSI phenotypic tumor subpopulation is characterized by tumors rich in immune cells and expressing neoantigens that activate antitumor immune responses [22]. Studies have shown that in patients with stage I, II, or III CRC, infiltration of specific functional immune cell subpopulations in tumors is associated with improved prognosis and a reduced risk of recurrence after surgery [23]. Immunotherapy may therefore provide clinical benefit for CRC patients, especially for advanced patients with very poor prognosis.

## Classification of T cells and antigen presentation mechanisms

### T-cell infiltration

Migration of effector T cells toward tumors is coordinated by interactions among chemokines, chemokine receptors, and adhesion molecules. Following activation, T cells are released from lymph nodes through downregulation of lymph node homing molecules such as CD62L and CCR7 [24], upregulation of sphingosine-1-phosphate receptor 1 (S1PR1) [25], and expression of other chemokine receptors and tissue-specific adhesion molecules [26]. These changes in adhesion molecule expression allow activated T cells to exit lymphoid tissues and migrate to peripheral sites.

T cells extravasate into tissues after passing through postcapillary venules [27]. Endothelial cells in the vasculature of peripheral tissues serve as crucial mediators of T-cell migration and extravasation into the TIME [28]. Interactions between selectins, such as E-selectin (CD62E) and P-selectin (CD62P), which are expressed on endothelial cells, and their respective ligands, which are expressed on effector T cells, result in slow rolling of T cells on the surface of blood vessels [29, 30]. The process of slow rolling further allows chemokine receptors on T cells to engage with chemokines secreted by endothelial cells. Binding of chemokine receptors leads to a conformational change in adhesion molecules from a low-affinity state to a high-affinity state, thereby enabling stronger adhesive interactions between T cells and the vascular system to support the trans-endothelial migration of effector T cells into the TIME [31].

### Antigen recognition

T-cell recognition is a multistep process that involves two steps. The first step is antigen presentation, where peptide antigens are presented on the cell surface in the form of either class I or class II human leukocyte antigens (HLAs) [32]. HLA-I is expressed on all nucleated cells, including tumor cells, and serves as a ligand for the TCR expressed on CD8 + cytotoxic T cells. In a state of homeostasis, HLA-I binds to “self” peptides, which derive from proteasomal degradation of old proteins or from ribosomal translational stalling products known as defective ribosomal products (DRiPs) [33]. Within the endoplasmic reticulum, they are loaded onto HLA-I molecules in a competitive manner facilitated by chaperone proteins, such as TAPASIN [34]. In infection or cancer, the mode of antigen presentation to the HLA-I pathway for pathogen-derived sequences or neoantigens is similar to that of “retired personnel” and “DRiP” forms of “self” peptides.

The ligand for TCRs expressed on CD4 + T cells is HLA-II, which is expressed on specialized antigen presenting cells (APCs) such as dendritic cells, monocytes/macrophages, and B cells. There is evidence that tumors directly express HLA-II [35], but it is generally believed that recognition of HLA-II-restricted neoantigens occurs through interactions with APCs [36]. HLA-II molecules bind peptides within the late endosomal compartment, intercepting endocytosed proteins that are protected from degradation by endosomal proteases [32]. Cochaperone proteins such as HLA-DMs facilitate the exchange of high-affinity peptides [37]. Neoantigens can enter this pathway through endocytic uptake of apoptotic or necrotic tumor cells harboring specific mutations. Once presented by HLA-I and HLA-II molecules, neoantigen peptides can be detected by T cells through their α-β T-cell receptors (αβTCRs).

The TCR-MHC signaling pathway is regulated by costimulatory or coinhibitory signals, which tumor cells exploit to evade destruction. Immune checkpoint inhibitors (ICIs) target coinhibitory receptors, such as cytotoxic T lymphocyte antigen 4 (CTLA-4) and programmed cell death protein 1 (PD-1), on T cells and other immune cell subsets or their ligands, such as programmed cell death ligand 1 (PD-L1), on tumor cells and various immune cells [38]. Consequently, ICIs prevent T-cell dysfunction and apoptosis, enhance T-cell activation, and augment the cytotoxic killing of tumor cells.

## Regulation of T cells in TIME

### Autophagy regulation in TIME

Autophagy is a key process that links cancer cells to TIME by secreting pro-inflammatory factors that regulate tumor growth, metastasis, and angiogenesis, as well as immune escape [39, 40]. Inhibition of autophagy in cancer cells is associated with reduced release of several cytokines and chemokines, which affected the T cell and dendritic cell recruitment, so immune surveillance escapes occur [5, 41].

As for the immune cell, autophagy induces abnormal protein degradation in tumor-specific CD8 + T cells and NK cells, allowing APCs to utilize these proteins as MHC-I and II [42]. In addition, immunosuppressive cells vary in their response to autophagy suppression. For example, the immunosuppressive effect of Tregs is highly autophagy dependent, and *ATG5* or *ATG7* deletion in T cells induces severe tumor implantation rejection in isogenic mouse tumor models [43]. On the other hand, some studies have shown that M1 macrophages can inhibit tumor progression. Autophagy is involved in the production and polarization of macrophages, and the deficiency of Toll-like receptor 2 (TLR2) is associated with the inhibition of autophagy, which subsequently leads to the biosynthesis of M2-type macrophages, thereby supporting tumor progression [44].

Autophagy also has an impact on immune tolerance of immunotherapy, for example, immune molecules such as PD-1 and CTLA-4 are regulated by the autophagy. PD-1 is an important molecule that inhibits anti-tumor immunity, and PD-1 has been reported to induce autophagy [45]. PD-L1 overexpressing cells responded more sensitively to autophagy inhibitors than cells with weaker PD-L1 expression, suggesting that autophagy inhibitors may be an important therapeutic method for PD-L1 overexpressing cancer cells. The CTLA-4 is another immune tolerance checkpoint that can be targeted to treat tumors. A cancer antigen called MAGE-A is associated with resistance to CTLA-4 inhibitors and inhibits autophagy, suggesting that autophagy induction could be used therapeutically to improve the efficacy of CTLA-4 inhibitors in tumors [46], further experiments are needed to explore the crosstalk of autophagy and immune checkpoints in CRC.

### Complements regulation in TIME

Complements act as a bridge between tumor progression and immune response. In CRC patients, the lectin pathway has been reported to be significantly enhanced compared to normal individuals [47]. Complements drive crosstalk between macrophages and cancer cells, and cytokine secretion from macrophages is regulated by the inhibition of complement like C5a [48]. In addition, in CRC, C5a-C5aR complement signal transduction pathway can activate the NF-κB pathway and the expression of transcription factor AP-1, which promotes the production of matrix metalloproteinases MMP-1 and MMP-9, then affects extracellular matrix decomposition and tumor progression [49, 50]. Moreover, C3, C3a-C3aR axis, C5a/C5aR1 axis and other complement pathways can also regulate immune cell response and TIME, and participate in tumor progression in CRC [51, 52]. Such changes in complements and pathways may be potential biomarkers for prognosis in CRC patients.

### Oxidative stress and TIME

Oxidative stress, and elevated ROS levels, are hallmarks of cancers. The physiological level of ROS is important for T cell activation, expansion and effector function [53]. In vitro T cell activation and in vivo antigen-specific T cell expansion require specific ROS-dependent signal transduction [54]. Enhanced oxidative stress can lead to the impairment of anti-tumor function of T cells [55]. On the other hand, increased ROS production induced by mitochondrial dysfunction drives T cell depletion, while ROS neutralization slows T cell terminal differentiation [56].

Oxidative stress can regulate immune cell function in a variety of ways. For example, ROS can epigenetically regulate the stability of the SUMO-specific protease 3 (SENP3) and maintain immune cells in TIME [57, 58]. What’s more, T cell intrinsic ROS is essential for metabolic adaptation and effector function of CD8 + T cells. Under the action of TCR-mediated ROS, SENP7 is rapidly translocated from the nucleus to the cytoplasm, promoting PTEN degradation, thus maintaining the metabolic state of CD8 + T cells in the TIME [58].

## T-cell-related immunotherapies in colorectal cancer

In recent years, tumor immunotherapy has achieved remarkable success in clinical applications. Cancer immunotherapy encompasses various strategies, such as the administration of cytokines to stimulate immune cells, monoclonal antibodies targeting immune checkpoint inhibitors, vaccines, and CAR-T-cell therapy.

### Administration of cytokines

**Fig. 1:**
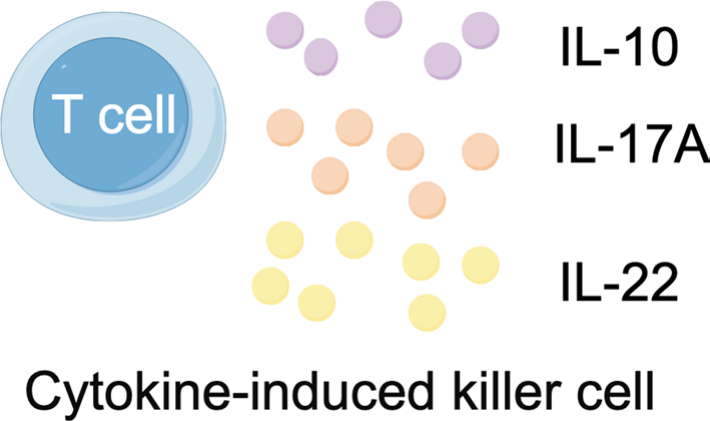
Administration of cytokines. Dysregulated inflammatory responses of T cell related cytokines (IL-10, IL-17A, IL-22 etc.) refers to the delivery for therapeutic purposes

Administration of cytokines refers to the delivery of these small proteins to the body for therapeutic purposes (Fig. 1). Direct inhibition of known effector cytokines that drive tumor progression may improve patient prognosis [9]. Inhibition of the suppressive cytokine IL-10 represents a potential target for triggering potent antitumor immunity. Serum IL-10 levels have been found to be positively associated with tumor stage and negatively with prognosis in CRC patients [59]. IL-10 is upregulated in the microenvironment of CRC, and levels of IL-10RA correlate with Ki67 staining [60]. An IL-10 blockade antibody drives accumulation of TILs, release of granzyme B, and tumor cell necrosis in an in vitro human CRC culture system. However, systemic blockade of IL-10 or IL-10RA poses significant risks. Targeted approaches may be necessary. In a mouse model of CRC, intra-tumoral injection of lentiviral vectors encoding IL-10 shRNA effectively reduced IL-10 expression and enhanced the efficacy of bone marrow-derived dendritic cell vaccines [61]. Given the role of Th17 and Th22 cells in promoting tumor development, IL-17A, IL-17F, and IL-22 are also promising targets in CRC. Depletion of Il17a or Il17f reduces tumor development in APC-driven mouse models of CRC. Blocking the IL-17/IL-17RA axis may also enhance the efficacy of anti-VEGF therapy. Anti-IL22 antibodies inhibit CRC cell proliferation in vitro. Gene therapy aimed at driving expression of IL-22BP, a secreted binding protein that inhibits IL-22 signaling, can alleviate tumor burden in mice. However, caution is needed, as some studies suggest that impairing the function of Th17 and Th22 cells promotes tumor occurrence and progression. The reasons for these divergent outcomes are not yet fully understood but may be related to the specific mechanisms underlying colorectal cancer pathogenesis and the role of T cells in promoting appropriate versus chronic, dysregulated inflammatory responses. Further elucidation of the roles of these cells in CRC is warranted.

### Checkpoint inhibitor

**Fig. 2:**
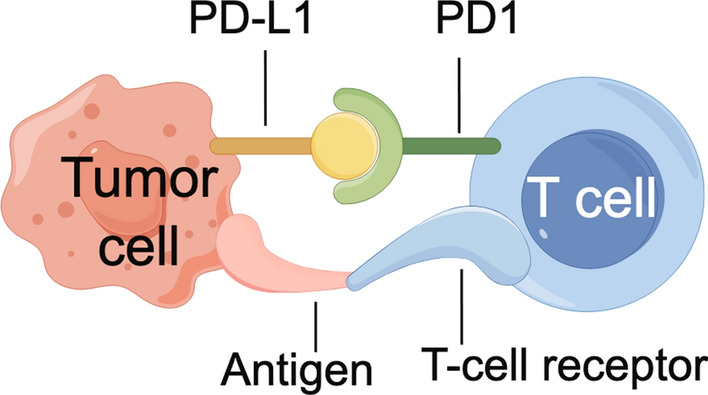
Mechanism diagram of ICI between tumor cells and T cell. Anti-PD-1 and PDL-1 monoclonal antibodies, are highly effective in CRC

ICI, such as anti-PD-1, PDL-1, and CTLA-4 monoclonal antibodies, are highly effective in CRC (Fig. 2). During tumor editing and immune evasion, tumor cells tend to express ligands for immune checkpoint receptors expressed on effector T cells, thereby dampening their immune function [62]. ICIs are a distinct type of immunotherapy that functions by inhibiting negative regulatory receptors on T cells, such as CTLA-4 and PD-1, thus enhancing the antitumor immune response. At present, biomarkers of ICI have been explored, regardless of the primary oringin, compared with mismatch repair ability (pMMR) or MSS tumors, tumors with dismatch repair-deficient (dMMR) or high levels of microsatellite instability (MSI-H) are highly responsive to ICI [63].

Immunotherapy of dMMR/MSI-H mCRC (Table 1): The U.S. Food and Drug Administration (FDA) approved pabolizumab and naboliumab in 2017 and approved the combination of naboliumab and ipilimumab in 2018 to treat chemotherapy-refractory dMMR/MSI-H mCRC. Pabolizumab has been shown to be effective against chemotherapy-resistant dMMR/MSI-H mCRC in multiple clinical trials. In the naboliumab monotherapy cohort, 74 dMMR/ MSIH mCRC patients who had failed to respond to at least one prior chemotherapy (including fluorouracil, oxaliplatin, and irinotican) received naboliumab monotherapy, and exactly 31% achieved the objective response as assessed by the investigators. 69% of patients got disease control for 12 weeks [64]. Grade 3 to 4 treatment-related adverse events (AE) occurred in 32% of patients. Elevated AST and/or ALT (11%), elevated lipase (4%), anemia (3%), and colitis (3%). 13% of patients stopped taking medication due to AE. Autoimmune hepatitis (2%) and acute kidney injury (2%). Some patients received immunomodulatory drugs to address these adverse events. The safety profile of these adverse events was manageable, and no treatment-related deaths were reported [65]. At present, it is difficult to directly compare the efficacy of pabolizumab, nebuliumab, and nebuliumab with ipilimumab.

**Table 1:** Clinical trials of immunotherapy for dMMR/MSI-H metastatic colorectal cancer

Trial	Agents	Target	Phase	ORR	mPFS(m)	mOS(m)
KEYNOTE-016	Pem	PD-1	II	40%	NR	NR
KEYNOTE-164	Pem	PD-1	II	33%	2.3	31.4
KEYNOTE-164	Pem	PD-1	II	33%	4.1	NR
ChckMate-142	Nivo	PD-1	II	31%	14.3	NR
ChckMate-142	Nivo + Ipi	PD-1/CTLA-4	II	55%	NR	NR
ChckMate-142	Nivo + Ipi	PD-1/CTLA-4	II	69%	NR	NR
KEYNOTE-177	Pem vs CT	PD-1	III	43.8% vs 33.1%	16.5 vs 8.2	NR

*dMMR* deficient mismatch repair, *MSI-H* microsatellite instability-high, *ORR* overall response rate, *mPFS* median progression free survival, *mOS* median overall survival, *Pem* pembrolizumab, Nivo nivolumab, *Ipi* ipilimumab, *CT* chemotherapy, *PD-1* programmed death-1, *CTLA-4* cytotoxic T-lymphocyte-associated antigen-4, *NR* not reported, vs versus

Immunotherapy for pMMR/MSS metastatic colorectal cancer (Table 2): The major subgroup of metastatic colorectal cancer is pMMR/MSS tumors, which account for 95% of all metastatic CRC. However, previous reports have shown that pMMR/MSS mCRC patients do not benefit clinically from ICI monotherapy [46, 66]. Various combination therapies with ICI have been explored to improve the immunogenic microenvironment and enhance the efficacy of ICI in pMMR/MSS metastatic CRC. At the same time, combination therapy with PD-1/PD-L1 and CTLA-4 blocking antibodies may provide synergistic anti-tumor benefits for dMMR/MSI-H mCRC patients [67].

**Table 2:** Clinical trials of immunotherapy for pMMR/MSS metastatic colorectal cancer

Trial	Agents	Target	Phase	ORR	mPFS(m)	mOS(m)
CCTG CO.26	Durvalumab + Tremelimumab **vs** BSC	PD-1/CTLA-4	II	DCR 23% vs 7%	1.8 vs 1.9	6.6 vs 4.1
REGONIVO EPOC1603	Nivolumab + regorafenib	PD-1 VEGFR	II	36%	7.9	12.3
BACCI	Atezolizumab + capecitabine + bevacizumab vs Capecitabine + bevacizumab + placebo	PD-1 VEGF	II	8.54% vs 4.35%	4.4 vs 3.3	NR
LCCC1632	Nivolumab + ipilimumab + panitumumab	PD-1 CTLA4 EGFR	II	35%	5.7	NR
NCT01988896	Atezolizumab + cobimetinib	PD-L1 MEK	Ib	8%	1.9	9.8
IMblaze370	Atezolizumab + cobimetinib vs Atezolizumab vs Regorafenib	PD-L1 MEK VEGFR	III	3% vs 2% vs 2%	1.91 vs 1.94 vs 2.00	8.518.87 vs 7.10 vs
NCT02437071	Pembrolizumab + radiotherapy	PD-L1	II	9%	NR	NR

*pMMR* proficient mismatch repair, *MSS* microsatellite stable, *ORR* overall response rate, *mPFS* median progression free survival, mOS median overall survival, *DCR* disease control rate, *Pem* pembrolizumab, *Nivo* nivolumab, *Ipi* ipilimumab, *PD-L1* programmed cell death ligand 1 *PD-1* programmed death-1, *CTLA-4* cytotoxic T-lymphocyte-associated antigen-4, *VEGFR* vascular endothelial growth factor receptor, *VEGF* vascular endothelial growth factor *MEK* Mitogen-Activated Protein Kinase Kinase, *vs* versus

Combination therapy: A randomized Phase III trial (KEYNOTE 177) compared pabolizumab monotherapy with 5-fluorouracil with or without bevacizumab or cetuximab in 307 previously untreated patients with dMMR/ MSIH CRC. Progression free survival (PFS) of pbolizumab was significantly longer than that of chemotherapy. Objective response rate (ORR) as a secondary endpoint was 43.8% in the pabolizumab group and 33.1% in the chemotherapy group. Grade 3 or higher treatment-related adverse events in the pabolizumab group compared to the chemotherapy group (22 and 66%, respectively) [68]. Based on these, in June 2020, the FDA approved Pabolizumab as a first-line treatment for dMMR patients with mCRC [69]. Moreover, in the first-line treatment cohort of the Phase II CheckMate 142 study, 45 patients with dMMR/MSI-H CRC who received naboliumab in combination with low-dose ipilimumab were observed to have benefits in PFS, ORR, and Overall survival rates (OS).

### Therapeutic aspects of the complement system in CRC

Complement activation is a key driver of multiple immune diseases. Some concerns have delayed the progress of complement-related therapies because they are known to block important branches of innate immunity. For example, complement inhibition impairs opsonism and bacteriolytic activity, thereby increasing the risk of infection. In the case of the use of ekuzumab, it has been found that although the use of the drug is effective, it is also associated with side effects, such as a high risk of meningococcal infection [70]. New generation of high-potential drugs is also progressing rapidly through clinical trials and could transform the field, as they will have the potential to inhibit complement beyond C5 in a variety of diseases and avoid the challenges associated with complement inhibition. Therefore, the next generation of complement-based therapies has the advantage of being safer, especially with a lower incidence of serious adverse reactions compared to older drugs [70].

The role of the complement system is still controversial, both pro-tumor and anti-tumor, and research suggests inhibiting complement activation as a novel strategy for cancer treatment, possibly using C5aR and C3aR blockers [49]. Anti-complement drugs are considered a potential approach in cancer treatment that can be used in combination with traditional chemotherapy or immune checkpoint inhibitors without increasing the side effects of chemotherapy [71]. What’s more, complements inhibition has a broad role in enhancing the efficacy of immunotherapy, especially when the complement receptors C3aR and C5aR are expressed on CD8 + T cell and genetically engineered T cells [72]. In addition, targeting the complement C3aR/C5aR/IL-10 pathway has been suggested in conjunction with other therapeutic modalities because it enhances the anti-tumor efficacy of T cells [73]. A previous study used a fusion protein (anti-PD-1-IL10) or (anti-CTLA4-IL-10) for CD8 + T cells amplification in adoptive cell therapy [72]. This synergistic effect was further tested in other studies in preclinical models of colon and lung cancer [74, 75], and paving the way for future clinical trials. However, the risks and benefits of anti-complement therapy in combination with other anti-cancer drugs should be further investigated.

Several studies have reported improvement of complement-mediated monoclonal antibody (mAb) effects through genetic engineering, coupling, and even glycosylation. Others have suggested converting non-complement-binding antibodies into complement-binding antibodies, such as IgG1 and IgG3, which are most effective at activating complement [76]. For example, Fc components can be designed to enhance the cytotoxic activity of therapeutic monoclonal antibodies, while bispecific antibodies can be designed to recruit complement effector functions, and alterations in glycosylation are found to improve the cleavage potential of monoclonal antibodies [77, 78].

Several studies have scrutinized the effects of treatment that blocks the complement system in mouse models. For example, complement depletion inhibits tumor growth in a mouse model of colon cancer [79]. In addition, when treating mice with these inhibitors, enhanced immune cell infiltration (CD8 + T cells) and increased chemokine expression were observed in TIME [79]. Since the C5a/C5aR1 signal transduction axis is known to play a role in TIME immune infiltration, a number of studies have explored the effect of inhibiting the C5a/C5aR1 axis on CRC. It was found that C5a/C5aR1 axis can alleviate the tumor progression, significantly inhibit pro-inflammatory factors, and induce the production of anti-inflammatory factors [52]. Also, other C5aR antagonists, including PMX53, were effective in reducing tumor size and enhancing the effects of anti-cancer chemotherapy in mice [75, 80]. It is worth mentioning that targeting the receptor C5aR, rather than the components C3 or C5, can cause opsonation to protect patients from the risk of bacterial infection. Targeting C5aR, however, leaves the other complement system component, C3a, uninhibited [80].

### Peptide vaccines

**Fig. 3:**
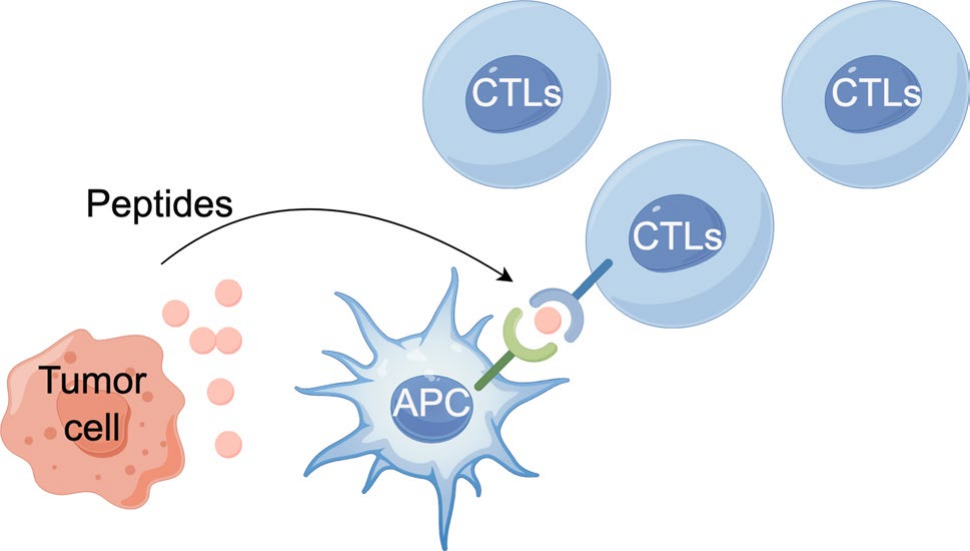
Peptide vaccines are promising tools for treatment of CRC. CRC related peptides, and the induced immune response between CTLs, APCs and tumor cells were significant

Peptide vaccines are promising tools for treatment of CRC **(**Fig. 3**)**. One of the earliest identified markers was carcinoembryonic antigen (CEA), which is also overexpressed in CRC [81]. However, since CEA is also expressed in normal epithelial cells, it is not truly cancer specific, and there is a need to develop CEA-altering peptide ligands with higher HLA binding affinity that can effectively activate specific CTLs in vitro [82]. Another way to increase CTL activation is to develop DNA vaccines that encode CEA-derived peptides and sequences for stimulating cytokines, adjuvants, or supportive T helper cell epitopes. In mouse models, the vaccine showed greater T-cell activation than did the pure peptide vaccine, but the results were still not satisfactory [83]. A recent study found that in MUC1.Tg mouse models, peptide vaccination consisting of two different 9-polymers derived from MUC-1 combined with CpG oligonucleotides and granulocyte–macrophage colony–stimulating factor (GM-CSF) as adjuvants reduced tumor load. In a prophylactic setting, complete protection against isogenic colon cancer cell lines was achieved through specific activation of the immune system by MUC1 [84].

Gradual acquisition and accumulation of mutations are important in the study of cancer occurrence and development. TGFβRII mutation-reactive T cells reduce tumor load in mouse models and significantly extend survival [85]. For example, in a clinical trial, 22 CRC patients were vaccinated with vaccines containing the frameshift AIM2, HT001, and TAF1B peptides, and the induced immune response was significant, with all patients responding to at least one peptide [3].

### CAR-T cells

**Fig. 4:**
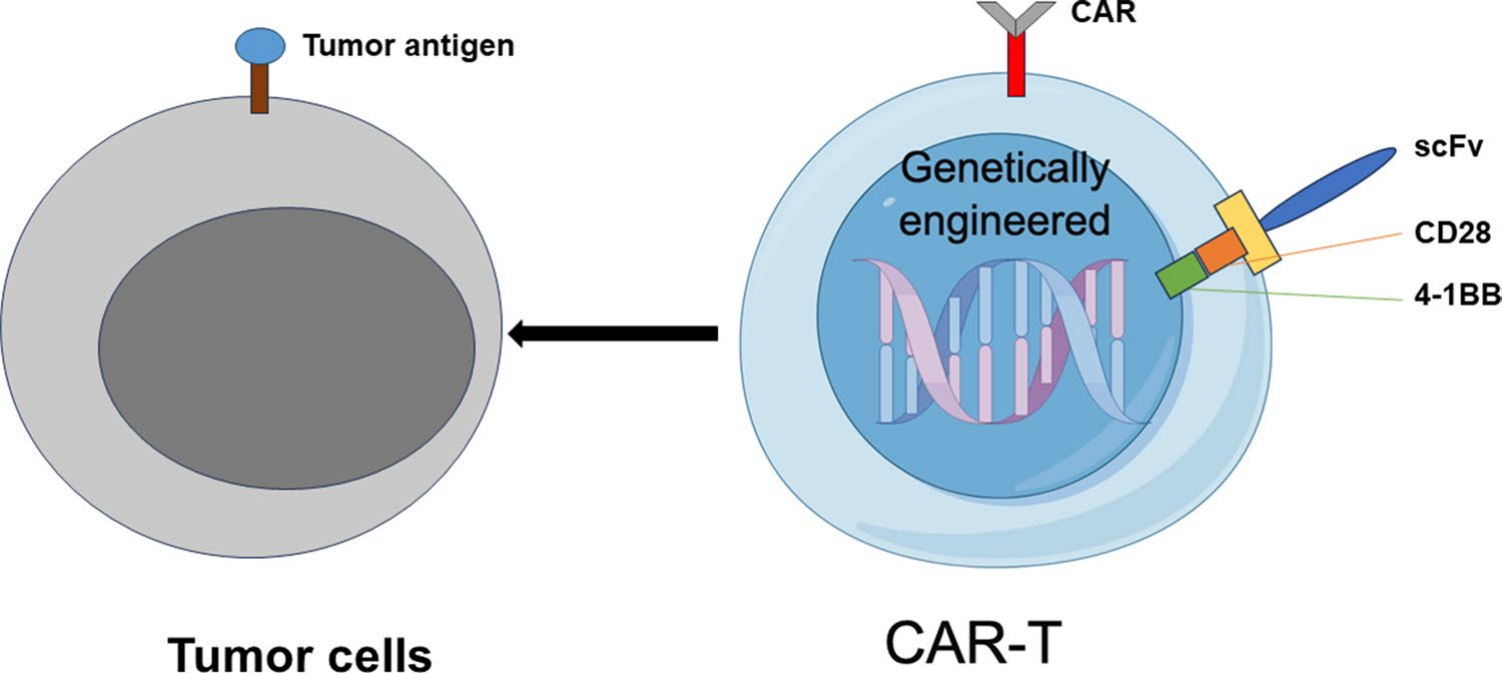
Chimeric antigen receptor (CAR) T-cell therapy is a new type of immunotherapy in which CAR-targeting molecules are expressed by genetically engineered T cells. CAR-T cells kill tumor cells by releasing inflammatory factors

Chimeric antigen receptor (CAR) T-cell therapy is a new type of immunotherapy in which CAR-targeting molecules are expressed by genetically engineered T cells (Fig. 4). CAR-T-cell therapy aims to introduce CAR-T cells into patient-derived peripheral blood T cells in a fairly short period of time, in which the T cells' biological properties are redirected and reprogrammed [86]. The T cells are then rapidly expanded in vitro to obtain memory and effector lymphocytes with high affinity. These T cells are subsequently injected into patients and robustly proliferate and induce potent antitumor activity. These synthetic receptors use single-stranded variable fragments (scFvs) from variable regions to recognize their corresponding specific antigens in a major histocompatibility complex-independent manner [87]. Most scFvs have binding properties similar to those of antibodies. Recent strategies have focused on harnessing CAR-T-cell therapy through engineered CARs, T cells, and interactions with other elements of the TIME. Among them, reshaping the TIME is one of the most attractive strategies for promoting endogenous immune responses to achieve permanent CAR-T-cell binding.

The process of selecting cancer-specific target antigens for CAR T cell therapy is a critical step in the development of this immunotherapy, which determines the specificity and safety of CAR T cell therapy [88]. Antigens should be highly uniformly expressed on the surface of cancer cells, while little or no expression on healthy tissues, and a larger antigen pool enhances the potential for a robust and sustained anti-cancer immune response, with stable target antigens helping to reduce the risk of recurrence [89]. Therefore, optimizing the selection of target antigens, which can not only improve the anti-tumor response, but also reduce the antigen escape mechanism to prevent recurrence, is the key to the success of CAR T therapy.

A key issue in developing CAR-T cell therapies for cancer treatment is identifying target antigens specific to each cancer type. MAGE-A, PASD1, NY-ESO-1, LAGE-1, OIP5, TTK, PLU1, DKKL1 and FBXO39 were all overexpressed in CRC [90, 91]. CEA is one of the most studied targets of anti-CRC CAR-T cells. In addition, second-generation CAR-T cells targeting CEA showed excellent anti-tumor effects both in vitro and in vivo, which were enhanced when combined with interleukins such as IL-12 [92]. Both second—and third-generation CAR-T cells were used for in vitro and in vivo studies. Tandem CAR-T cells targeting CEA and CD30 showed significantly increased cytotoxicity, persistence and release of perforin and granzyme B compared to CEA-CAR T cells. Similarly, CD30/TAG72-CAR T cells showed increased cytotoxicity compared to TAG72-CAR T cells. In contrast, tandem CAR-T cells that were anti-CEA and CD25 showed higher cell persistence compared to anti-CEA-CAR T cells, but had similar cytotoxicity to CRC models [93].

With respect to colorectal cancer, several clinical trials have been approved for multiple antitumor antigens. Most trials are in the early stages, phase I or I/II. The most commonly studied targets for CRC in CAR-T cell therapy are CEA and NKG2DL, followed by EGFR and HER2. The first CAR-T clinical trial for CRC, which began in 2014, studied the safety and efficacy of second-generation CEA-CAR T cells in patients with CRC, and also included patients with lung, stomach, breast, and pancreatic cancers (NCT02349724) [87]. There are only two allogeneic CAR-T cell therapies for CRC in clinical trials (NCT04107142 and NCT03692429), which recently began in 2019 and 2020, respectively [87]. Both CAR T cell therapies target NKG2D ligands, but differ in the strategies used to make them suitable for allogeneic use. The first specifically used gamma-delta T cells as a source for making allogeneic CAR-T cells [94]. Their tissue-resident mode makes them more susceptible to infection by non-inflammatory tumors, and they are also able to recognize multiple tumor antigens through their innate cytotoxic receptors, thereby reducing immune escape. The second CAR T cell product uses a non-gene editing strategy based on the TCR suppressor molecule (TIM) sequence contained in the construct. Therefore, when the peptide is expressed, it interferes with endogenous TCR signaling and controls GvHD. Although further research is needed, preclinical and clinical data suggest that CAR-T cell therapy is a promising strategy for treating colorectal cancer, even in advanced stages.

## Limitations and prospects

There are limitations to T-cell-targeted immunotherapy for colorectal cancer patients. First, different molecular subtypes of CRC may play crucial roles in determining the success of certain treatment approaches. Due to the heterogeneity of CRC, stratification of treatment based on tumor subtypes may be needed. Additionally, a better understanding of the (sub)phenotypic and functional diversity of T cells is still needed. This approach is crucial for preventing immune hemostasis impairment and unnecessary side effects.

A deeper understanding of the intersection between CD4 + T cells and CRC is also needed. What lies behind the seemingly contradictory roles played by certain cells? For example, both nTregs and pTregs contribute to controlling inflammation as lesions in CRC but become detrimental once inflammation leads to cancer. However, some Tregs escape their suppressive role and become eTregs, which participate in anticancer immune responses similar to those of effector cells. Similarly, Th17 and Th22 cells promote pathogen clearance and epithelial barrier function, respectively. Effective clearance and barrier integrity minimize epithelial cell exposure to harmful inflammatory stimuli. However, sustained activity of these cells promotes tumor development. In contrast, Th17 cells expressing TBET are an integral component of anticancer responses. Similarly, the interplay between follicular T cells and the colonic microbiota can either promote or counteract CRC. It is essential to address in more detail the development of these populations and their impact on CRC inflammatory responses to harness them for improving disease outcomes.

Focusing on the driver mutations responsible for maintaining the state of transformation and/or progression of an individual tumor is also one of the priorities of tumor immunotherapy research. Targeting driver mutations has advantages over targeting passenger mutations because the survival of tumor cells depends on these dysregulated gene products, and thus immune escape by turning off or restoring such mutations is less likely. The concept of personalized peptide vaccination follows a different approach. In addition to determining the HLA profile, CRC patients are screened for the presence of CTLs and IgG, which target known tumor-associated antigens and neoantigens. After understanding the HLA layout of the patient, as well as the natural immunogenicity of the tumor, the vaccine can be adjusted by selecting a matching peptide for vaccination. This reduces the risk of severe adverse events simply by enhancing the existing antitumor immune response.

## Conclusion

Tumor immune microenvironment plays an important role in tumor genesis and metastasis. Further investigation of TIME can not only reveal the mechanism of tumor progression, but also provide more targets for immunotherapy. Having more alternative and effective treatments available for treating patients with colorectal cancer is critical. Immunotherapy is a new option for cancer treatment. However, further studies on CRC-specific tumor antigens, the genetic regulation of T cells, and the regulation of immune activity are still necessary.

## Data Availability

Not applicable.
